# Supplementary material for: Predictors of Human Milk Fatty Acids and Associations with Infant Growth in a Norwegian Birth Cohort
Source: Nutrients. 2022 Sep 17;14(18):3858. doi: 10.3390/nu14183858 (PMC9503921; doi:10.3390/nu14183858)
Supplement: Supplementary file 1 [file nutrients-14-03858-s001.zip › nutrients-1869659-supplementary.pdf]

Supplemental Table S1. Adjustment sets for each predictor-triglyceride fatty acid composition model[1]

Predictor	Adjustment set
Body mass index (BMI)[2]	Maternal age, education[3], parity, infant age at milk collection[4], formula introduction[5]
Excess weight gain in pregnancy[6]	Gestational age, maternal age, education, parity, infant age at milk collection, formula introduction
Parity[7]	Maternal age, education, infant age at milk collection, formula introduction
Smoking[8]	Maternal education, infant age at milk collection, formula introduction
Gestational age[9]	BMI, smoking, parity, infant age at milk collection, formula introduction
Fatty fish intake[10]	BMI, education, cod liver oil, infant age at milk collection, formula introduction
Delivery mode[11]	Maternal age, BMI, birthweight, gestational age, excess weight gain in pregnancy, parity, smoking, infant age at milk collection, formula introduction
Cod liver oil intake[12]	Education, BMI, fatty fish intake, infant age at milk collection, formula introduction
Maternal age[13]	Parity, infant age at milk collection, formula introduction

[1] Outcome for all models was percent composition of triglyceride fatty acids in milk.
[2] Defined as underweight (BMI < 18.5 kg/m^2), normal/overweight weight (BMI 18.5 – 29.99 kg/m^2), or overweight/obese (BMI $\geq$30 kg/m^2).
[3] Defined as <12 years, 12 years, or >12 years of maternal education at the start of pregnancy.
[4] Measured in days.
[5] Defined as any formula consumed by the infant at the time that milk was collected (yes versus no) as reported by the mother.
[6] Defined by BMI according to guidelines set forth by Institutes of Medicine: for underweight women, >18.1kg; for normal weight women, >15.9kg; for overweight women, >11.3kg; and for obese women, >9.1kg.
[7] Defined as primiparous or multiparous.
[8] Current smoker versus former/never smoker at the start of pregnancy.
[9] Defined as gestational age at birth in days.
[10] Defined as number of fatty fish dinners consumed in the previous year.
[11] Vaginal delivery versus cesarean section.
[12] Defined as servings of cod liver oil consumed in the previous year.
[13] Defined as maternal age in years at the start of pregnancy.

Supplemental Figure S1. Directed acyclic graph representing association between triglyceide fatty acid composition in milk and infant growth between 0 and 6 months
Legend: Green circle represents exposure and its antecedents, blue circle represents outcome and its antecedents. Red circles represent confounding variables. Arrows represent direction of effect.

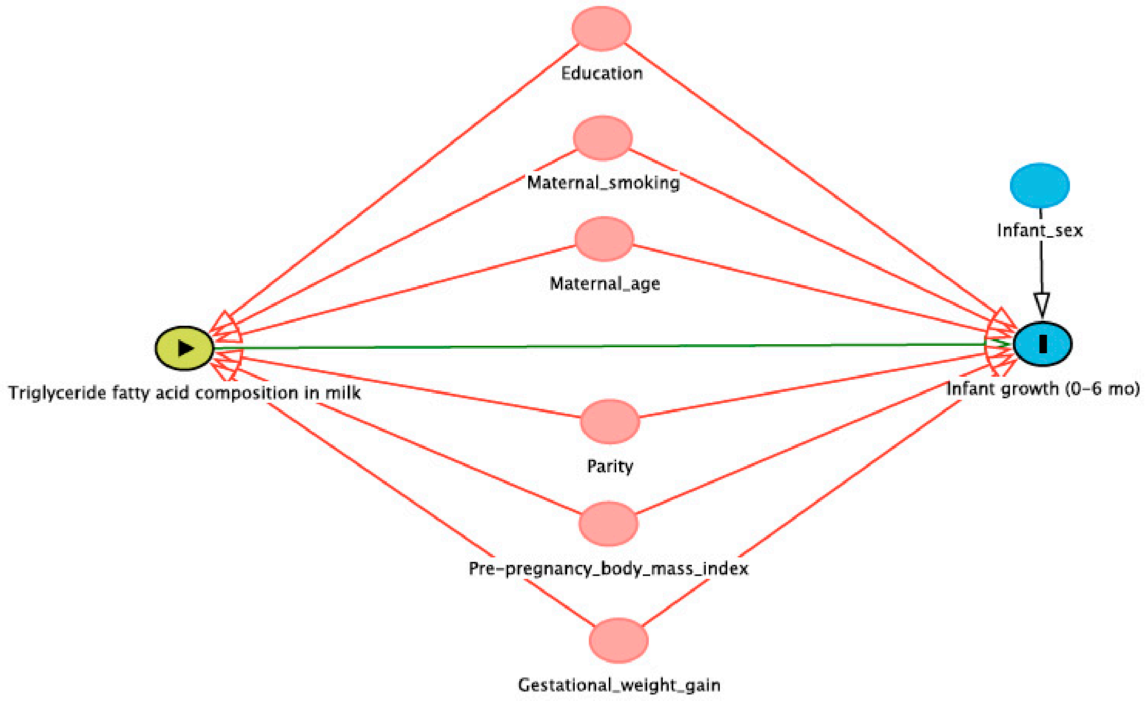

Supplemental Table S2. Comparison of study subset ($n = 789$) to remaining HUMIS-NoMIC cohort ($n = 1817$) and larger Norwegian population

Characteristic[1]	Study subset	Participants not included		General population[2]
	n=789	n=1817	p-value[3]	n=126182
Maternal age (years)	29.6	30.1	*0.02*	29
Childs birth weight (g)	3629	3479	*<0.001*	3570
Gestational age (days)	280	277	*<0.001*	282
Maternal parity (%)			*0.07*	
Primiparous	39.8	43.64		43.8
Maternal smoking[4] (%)			*0.93*	
No	88.4	87.4		86.8
Yes	11.6	12.6		13.2
Child sex (%)			*0.47*	
Female	53.5	52.0		48.7
Male	46.5	48.0		51.2

[1] Mean value, unless otherwise noted.
[2] Information on the general population of recent mothers was obtained through the Norwegian Medical Birth Registry (which records all births in Norway). Mothers who had given birth between 2001 and 2003 were selected and their characteristics compared to the HUMIS participants.
[3] Differences between study sample, remaining HUMIS participants evaluated through the student's t-test for continuous variables and chi-square test for categorical variables.
[4] Smoking at the start of pregnancy.

Supplemental Table S3. Associations[1] of maternal predictors and triglyceride fatty acid percent composition

Exposure	Outcome[2]	β-Coefficient	P-value[3]	L95[4]	U95[5]
Underweight body mass index (BMI)[6]	C8:0	0.03	0.15	-0.01	0.06
Underweight BMI	C10:0	-0.01	0.84	-0.14	0.12
Underweight BMI	C12:0	0.09	0.75	-0.49	0.68
Underweight BMI	C13:0	0.03	0.003	0.01	0.05
Underweight BMI	C14:0	0.18	0.49	-0.33	0.68
Underweight BMI	C14:1	-0.03	0.003	-0.06	-0.01
Underweight BMI	C15:0	0.01	0.57	-0.02	0.04
Underweight BMI	C15:1	0.00	0.27	-0.01	0.00
Underweight BMI	C16:0	-0.09	0.77	-0.67	0.49
Underweight BMI	C16:1t	-0.01	0.10	-0.02	0.00
Underweight BMI	C16:1n-7	-0.30	0.005	-0.51	-0.09
Underweight BMI	C17:0	-0.01	0.27	-0.02	0.01
Underweight BMI	C18:0	0.29	0.20	-0.16	0.74
Underweight BMI	C18:1t	-0.06	0.06	-0.13	0.00
Underweight BMI	C18:1n-9	-0.49	0.30	-1.41	0.43
Underweight BMI	C18:1n-7	-0.07	0.08	-0.15	0.01
Underweight BMI	C18:2n-6	0.50	0.26	-0.37	1.37
Underweight BMI	C18:3n-6	-0.02	0.006	-0.03	-0.01
Underweight BMI	C18:3n-3	-0.01	0.87	-0.11	0.09
Underweight BMI	C20:1n-9	0.01	0.75	-0.03	0.04
Underweight BMI	C20:2n-6	0.03	0.25	-0.02	0.08
Underweight BMI	C20:3n-9	0.00	0.73	0.00	0.00
Underweight BMI	C20:3n-6	-0.02	0.25	-0.04	0.01
Underweight BMI	C20:4n-6	-0.02	0.09	-0.05	0.00
Underweight BMI	C20:3n-3	0.00	0.09	0.00	0.01
Underweight BMI	C22:0	0.02	0.03	0.00	0.03
Underweight BMI	C22:1t	-0.01	0.27	-0.02	0.01
Underweight BMI	C20:5n-3	-0.01	0.55	-0.03	0.02
Underweight BMI	C22:4n-6	0.00	0.74	-0.01	0.01
Underweight BMI	C22:5n-6	0.01	0.02	0.00	0.01
Underweight BMI	C22:5n-3	-0.01	0.41	-0.03	0.01
Underweight BMI	C22:6n-3	-0.03	0.43	-0.11	0.05
Underweight BMI	SFA	0.54	0.42	-0.77	1.84
Underweight BMI	PUFA	0.43	0.39	-0.56	1.41

**Exposure**	**Outcome**[2]	**β-Coefficient**	**P-value**[3]	**L95**[4]	**U95**[5]
Underweight BMI	n-6 PUFA	0.48	0.30	-0.42	1.39
Underweight BMI	n-3 PUFA	-0.05	0.55	-0.22	0.12
Underweight BMI	MUFA	-0.89	0.09	-1.92	0.14
Underweight BMI	Trans FA	-0.08	0.03	-0.15	-0.01
Underweight BMI	SFA/PUFA	-0.03	0.86	-0.31	0.26
Underweight BMI	n-6/n-3 PUFA	0.48	0.15	-0.17	1.12
Underweight BMI	MCFA	0.28	0.64	-0.89	1.46
Underweight BMI	LC-PUFA	-0.08	0.26	-0.21	0.06
Obese BMI[7]	C8:0	0.00	0.91	-0.02	0.02
Obese BMI	C10:0	0.02	0.63	-0.06	0.10
Obese BMI	C12:0	0.16	0.38	-0.19	0.51
Obese BMI	C13:0	0.00	0.42	-0.02	0.01
Obese BMI	C14:0	0.15	0.33	-0.15	0.45
Obese BMI	C14:1	-0.02	0.01	-0.03	0.00
Obese BMI	C15:0	-0.04	<0.001	-0.06	-0.02
Obese BMI	C15:1	-0.01	0.01	-0.01	0.00
Obese BMI	C16:0	0.15	0.42	-0.21	0.50
Obese BMI	C16:1t	-0.01	0.04	-0.01	0.00
Obese BMI	C16:1n-7	0.00	1.00	-0.13	0.13
Obese BMI	C17:0	-0.02	<0.001	-0.03	-0.01
Obese BMI	C18:0	-0.19	0.16	-0.46	0.08
Obese BMI	C18:1t	-0.06	0.003	-0.10	-0.02
Obese BMI	C18:1n-9	0.43	0.12	-0.11	0.97
Obese BMI	C18:1n-7	0.03	0.21	-0.02	0.08
Obese BMI	C18:2n-6	-0.37	0.17	-0.90	0.16
Obese BMI	C18:3n-6	-0.01	0.21	-0.01	0.00
Obese BMI	C18:3n-3	-0.06	0.05	-0.12	0.00
Obese BMI	C20:1n-9	-0.02	0.06	-0.04	0.00
Obese BMI	C20:2n-6	0.01	0.43	-0.02	0.04
Obese BMI	C20:3n-9	0.00	0.90	0.00	0.00
Obese BMI	C20:3n-6	0.01	0.12	0.00	0.03
Obese BMI	C20:4n-6	-0.01	0.46	-0.02	0.01
Obese BMI	C20:3n-3	0.00	0.51	0.00	0.00
Obese BMI	C22:0	0.00	0.96	-0.01	0.01
Obese BMI	C22:1t	-0.01	0.02	-0.02	0.00
Obese BMI	C20:5n-3	-0.03	<0.001	-0.05	-0.02

Exposure	Outcome[2]	β-Coefficient	P-value[3]	L95[4]	U95[5]
Obese BMI	C22:4n-6	0.01	0.06	0.00	0.01
Obese BMI	C22:5n-6	0.00	0.28	-0.01	0.00
Obese BMI	C22:5n-3	-0.02	<0.001	-0.03	-0.01
Obese BMI	C22:6n-3	-0.10	<0.001	-0.15	-0.06
Obese BMI	SFA	0.22	0.57	-0.55	1.00
Obese BMI	PUFA	-0.57	0.06	-1.16	0.03
Obese BMI	n-6 PUFA	-0.35	0.21	-0.90	0.20
Obese BMI	n-3 PUFA	-0.22	<0.001	-0.32	-0.11
Obese BMI	MUFA	0.42	0.18	-0.19	1.03
Obese BMI	Trans FA	-0.08	0.001	-0.12	-0.03
Obese BMI	SFA/PUFA	0.17	0.05	0.00	0.34
Obese BMI	n-6/n-3 PUFA	0.66	0.001	0.28	1.05
Obese BMI	MCFA	0.33	0.36	-0.38	1.03
Obese BMI	LC-PUFA	-0.14	<0.001	-0.22	-0.06
Excess weight gain in pregnancy[8]	C8:0	-0.01	0.11	-0.03	0.00
Excess weight gain in pregnancy	C10:0	-0.08	0.006	-0.13	-0.02
Excess weight gain in pregnancy	C12:0	-0.32	0.01	-0.57	-0.07
Excess weight gain in pregnancy	C13:0	-0.01	0.10	-0.02	0.00
Excess weight gain in pregnancy	C14:0	-0.26	0.02	-0.47	-0.05
Excess weight gain in pregnancy	C14:1	0.01	0.18	0.00	0.02
Excess weight gain in pregnancy	C15:0	-0.01	0.18	-0.02	0.00
Excess weight gain in pregnancy	C15:1	0.00	0.41	0.00	0.00
Excess weight gain in pregnancy	C16:0	0.02	0.85	-0.23	0.27
Excess weight gain in pregnancy	C16:1t	0.00	0.38	0.00	0.01
Excess weight gain in pregnancy	C16:1n-7	0.27	<0.001	0.19	0.36
Excess weight gain in pregnancy	C17:0	0.00	0.28	-0.01	0.00
Excess weight gain in pregnancy	C18:0	-0.42	<0.001	-0.61	-0.23
Excess weight gain in pregnancy	C18:1t	0.01	0.61	-0.02	0.04
Excess weight gain in pregnancy	C18:1n-9	0.43	0.03	0.05	0.81
Excess weight gain in pregnancy	C18:1n-7	0.10	<0.001	0.07	0.13
Excess weight gain in pregnancy	C18:2n-6	0.26	0.16	-0.11	0.64
Excess weight gain in pregnancy	C18:3n-6	0.00	0.15	0.00	0.01
Excess weight gain in pregnancy	C18:3n-3	0.02	0.49	-0.03	0.06
Excess weight gain in pregnancy	C20:1n-9	0.00	0.60	-0.02	0.01
Excess weight gain in pregnancy	C20:2n-6	0.00	0.93	-0.02	0.02
Excess weight gain in pregnancy	C20:3n-9	0.00	0.99	0.00	0.00

Exposure	Outcome[2]	β-Coefficient	P-value[3]	L95[4]	U95[5]
Excess weight gain in pregnancy	C20:3n-6	-0.01	0.17	-0.02	0.00
Excess weight gain in pregnancy	C20:4n-6	0.01	0.02	0.00	0.02
Excess weight gain in pregnancy	C20:3n-3	0.00	0.89	0.00	0.00
Excess weight gain in pregnancy	C22:0	-0.01	0.002	-0.02	0.00
Excess weight gain in pregnancy	C22:1t	0.00	0.16	-0.01	0.00
Excess weight gain in pregnancy	C20:5n-3	0.00	0.66	-0.01	0.01
Excess weight gain in pregnancy	C22:4n-6	0.00	0.20	0.00	0.01
Excess weight gain in pregnancy	C22:5n-6	0.00	0.90	0.00	0.00
Excess weight gain in pregnancy	C22:5n-3	0.00	0.51	-0.01	0.01
Excess weight gain in pregnancy	C22:6n-3	0.00	0.94	-0.03	0.03
Excess weight gain in pregnancy	SFA	-1.10	<0.001	-1.64	-0.55
Excess weight gain in pregnancy	PUFA	0.29	0.18	-0.13	0.71
Excess weight gain in pregnancy	n-6 PUFA	0.28	0.16	-0.11	0.66
Excess weight gain in pregnancy	n-3 PUFA	0.01	0.76	-0.06	0.08
Excess weight gain in pregnancy	MUFA	0.80	<0.001	0.37	1.23
Excess weight gain in pregnancy	Trans FA	0.01	0.73	-0.03	0.04
Excess weight gain in pregnancy	SFA/PUFA	-0.14	0.02	-0.26	-0.02
Excess weight gain in pregnancy	n-6/n-3 PUFA	0.12	0.41	-0.16	0.40
Excess weight gain in pregnancy	MCFA	-0.67	0.009	-1.17	-0.17
Excess weight gain in pregnancy	LC-PUFA	0.00	0.93	-0.06	0.06
Parity[9]	C8:0	-0.01	0.41	-0.02	0.01
Parity	C10:0	-0.08	0.005	-0.14	-0.02
Parity	C12:0	-0.17	0.20	-0.43	0.09
Parity	C13:0	0.00	0.93	-0.01	0.01
Parity	C14:0	0.01	0.93	-0.21	0.23
Parity	C14:1	0.01	0.31	-0.01	0.02
Parity	C15:0	0.00	0.62	-0.01	0.02
Parity	C15:1	0.00	0.19	0.00	0.01
Parity	C16:0	0.31	0.03	0.04	0.58
Parity	C16:1t	0.00	0.52	-0.01	0.00
Parity	C16:1n-7	-0.19	<0.001	-0.28	-0.09
Parity	C17:0	0.01	0.09	0.00	0.01
Parity	C18:0	0.50	<0.001	0.30	0.71
Parity	C18:1t	0.00	0.96	-0.03	0.03
Parity	C18:1n-9	-0.23	0.27	-0.63	0.18
Parity	C18:1n-7	-0.07	<0.001	-0.11	-0.04

Exposure	Outcome[2]	β-Coefficient	P-value[3]	L95[4]	U95[5]
Parity	C18:2n-6	0.02	0.90	-0.37	0.42
Parity	C18:3n-6	0.01	0.001	0.00	0.02
Parity	C18:3n-3	0.02	0.38	-0.03	0.07
Parity	C20:1n-9	-0.02	0.005	-0.04	-0.01
Parity	C20:2n-6	-0.02	0.14	-0.04	0.01
Parity	C20:3n-9	0.00	0.76	0.00	0.00
Parity	C20:3n-6	0.01	0.04	0.00	0.03
Parity	C20:4n-6	-0.01	0.33	-0.02	0.01
Parity	C20:3n-3	0.00	0.42	0.00	0.00
Parity	C22:0	0.01	0.05	0.00	0.02
Parity	C22:1t	0.00	0.53	-0.01	0.00
Parity	C20:5n-3	-0.02	<0.001	-0.04	-0.01
Parity	C22:4n-6	0.00	0.28	0.00	0.01
Parity	C22:5n-6	0.00	0.10	0.00	0.00
Parity	C22:5n-3	-0.02	0.001	-0.03	-0.01
Parity	C22:6n-3	-0.08	<0.001	-0.12	-0.05
Parity	SFA	0.58	0.05	0.00	1.16
Parity	PUFA	-0.07	0.75	-0.52	0.38
Parity	n-6 PUFA	0.03	0.89	-0.38	0.44
Parity	n-3 PUFA	-0.10	0.01	-0.18	-0.02
Parity	MUFA	-0.51	0.03	-0.96	-0.05
Parity	Trans FA	0.00	0.89	-0.03	0.03
Parity	SFA/PUFA	0.08	0.26	-0.06	0.21
Parity	n-6/n-3 PUFA	0.39	0.01	0.09	0.70
Parity	MCFA	-0.25	0.35	-0.77	0.27
Parity	LC-PUFA	-0.11	0.001	-0.17	-0.05
Smoking[10]	C8:0	0.01	0.23	-0.01	0.04
Smoking	C10:0	0.11	0.01	0.02	0.19
Smoking	C12:0	0.53	0.008	0.14	0.91
Smoking	C13:0	-0.01	0.15	-0.02	0.00
Smoking	C14:0	0.31	0.06	-0.01	0.64
Smoking	C14:1	0.00	0.97	-0.02	0.02
Smoking	C15:0	-0.02	0.12	-0.04	0.00
Smoking	C15:1	0.00	0.14	-0.01	0.00
Smoking	C16:0	-0.07	0.72	-0.47	0.33
Smoking	C16:1t	0.00	0.85	-0.01	0.01

Exposure	Outcome[2]	β-Coefficient	P-value[3]	L95[4]	U95[5]
Smoking	C16:1n-7	-0.11	0.14	-0.26	0.04
Smoking	C17:0	-0.01	0.14	-0.02	0.00
Smoking	C18:0	0.26	0.11	-0.06	0.57
Smoking	C18:1t	0.03	0.29	-0.02	0.07
Smoking	C18:1n-9	-0.56	0.06	-1.15	0.03
Smoking	C18:1n-7	-0.05	0.06	-0.11	0.00
Smoking	C18:2n-6	-0.15	0.60	-0.74	0.43
Smoking	C18:3n-6	0.00	0.57	-0.01	0.01
Smoking	C18:3n-3	-0.01	0.67	-0.08	0.05
Smoking	C20:1n-9	-0.04	0.007	-0.06	-0.01
Smoking	C20:2n-6	-0.01	0.45	-0.05	0.02
Smoking	C20:3n-9	0.00	0.53	0.00	0.00
Smoking	C20:3n-6	0.00	0.87	-0.02	0.02
Smoking	C20:4n-6	-0.01	0.10	-0.03	0.00
Smoking	C20:3n-3	0.00	0.25	-0.01	0.00
Smoking	C22:0	0.00	0.72	-0.01	0.01
Smoking	C22:1t	-0.01	0.05	-0.02	0.00
Smoking	C20:5n-3	-0.03	0.003	-0.05	-0.01
Smoking	C22:4n-6	0.00	0.33	-0.01	0.00
Smoking	C22:5n-6	0.00	0.72	0.00	0.00
Smoking	C22:5n-3	-0.03	<0.001	-0.05	-0.02
Smoking	C22:6n-3	-0.10	<0.001	-0.15	-0.04
Smoking	SFA	1.11	0.01	0.27	1.96
Smoking	PUFA	-0.36	0.28	-1.02	0.30
Smoking	n-6 PUFA	-0.19	0.54	-0.79	0.42
Smoking	n-3 PUFA	-0.17	0.006	-0.29	-0.05
Smoking	MUFA	-0.77	0.03	-1.45	-0.09
Smoking	Trans FA	0.02	0.51	-0.03	0.07
Smoking	SFA/PUFA	0.15	0.12	-0.04	0.34
Smoking	n-6/n-3 PUFA	0.58	0.02	0.10	1.06
Smoking	MCFA	0.96	0.01	0.19	1.72
Smoking	LC-PUFA	-0.17	<0.001	-0.27	-0.08
Gestational age[11]	C8:0	0.00	0.05	0.00	0.00
Gestational age	C10:0	0.00	0.002	-0.01	0.00
Gestational age	C12:0	-0.01	0.002	-0.02	-0.01
Gestational age	C13:0	0.00	0.88	0.00	0.00

Exposure	Outcome[2]	β-Coefficient	P-value[3]	L95[4]	U95[5]
Gestational age	C14:0	-0.02	<0.001	-0.02	-0.01
Gestational age	C14:1	0.00	0.99	0.00	0.00
Gestational age	C15:0	0.00	0.30	0.00	0.00
Gestational age	C15:1	0.00	0.03	0.00	0.00
Gestational age	C16:0	0.00	0.72	-0.01	0.01
Gestational age	C16:1t	0.00	0.34	0.00	0.00
Gestational age	C16:1n-7	0.01	0.002	0.00	0.01
Gestational age	C17:0	0.00	0.15	0.00	0.00
Gestational age	C18:0	0.00	0.38	-0.01	0.00
Gestational age	C18:1t	0.00	0.41	0.00	0.00
Gestational age	C18:1n-9	0.02	0.001	0.01	0.04
Gestational age	C18:1n-7	0.00	0.01	0.00	0.00
Gestational age	C18:2n-6	0.01	0.45	-0.01	0.02
Gestational age	C18:3n-6	0.00	0.10	0.00	0.00
Gestational age	C18:3n-3	0.00	0.67	0.00	0.00
Gestational age	C20:1n-9	0.00	0.80	0.00	0.00
Gestational age	C20:2n-6	0.00	0.08	0.00	0.00
Gestational age	C20:3n-9	0.00	0.63	0.00	0.00
Gestational age	C20:3n-6	0.00	0.06	0.00	0.00
Gestational age	C20:4n-6	0.00	0.43	0.00	0.00
Gestational age	C20:3n-3	0.00	0.01	0.00	0.00
Gestational age	C22:0	0.00	0.43	0.00	0.00
Gestational age	C22:1t	0.00	0.05	0.00	0.00
Gestational age	C20:5n-3	0.00	0.96	0.00	0.00
Gestational age	C22:4n-6	0.00	0.97	0.00	0.00
Gestational age	C22:5n-6	0.00	0.57	0.00	0.00
Gestational age	C22:5n-3	0.00	0.52	0.00	0.00
Gestational age	C22:6n-3	0.00	0.50	0.00	0.00
Gestational age	SFA	-0.03	0.001	-0.05	-0.02
Gestational age	PUFA	0.00	0.66	-0.01	0.02
Gestational age	n-6 PUFA	0.00	0.54	-0.01	0.02
Gestational age	n-3 PUFA	0.00	0.48	0.00	0.00
Gestational age	MUFA	0.03	<0.001	0.02	0.05
Gestational age	Trans FA	0.00	0.58	0.00	0.00
Gestational age	SFA/PUFA	0.00	0.18	-0.01	0.00
Gestational age	n-6/n-3 PUFA	0.01	0.26	0.00	0.02

**Exposure**	**Outcome[2]**	**β-Coefficient**	**P-value[3]**	**L95[4]**	**U95[5]**
Gestational age	MCFA	-0.03	<0.001	-0.05	-0.02
Gestational age	LC-PUFA	0.00	0.40	0.00	0.00
Fatty fish intake[12]	C8:0	0.00	0.18	0.00	0.00
Fatty fish intake	C10:0	0.00	0.83	0.00	0.00
Fatty fish intake	C12:0	0.00	0.58	-0.01	0.00
Fatty fish intake	C13:0	0.00	0.73	0.00	0.00
Fatty fish intake	C14:0	0.00	0.72	0.00	0.00
Fatty fish intake	C14:1	0.00	0.30	0.00	0.00
Fatty fish intake	C15:0	0.00	0.21	0.00	0.00
Fatty fish intake	C15:1	0.00	0.83	0.00	0.00
Fatty fish intake	C16:0	0.00	0.17	-0.01	0.00
Fatty fish intake	C16:1t	0.00	0.17	0.00	0.00
Fatty fish intake	C16:1n-7	0.00	0.03	0.00	0.00
Fatty fish intake	C17:0	0.00	0.13	0.00	0.00
Fatty fish intake	C18:0	0.00	0.77	0.00	0.00
Fatty fish intake	C18:1t	0.00	0.32	0.00	0.00
Fatty fish intake	C18:1n-9	0.01	0.09	0.00	0.01
Fatty fish intake	C18:1n-7	0.00	0.77	0.00	0.00
Fatty fish intake	C18:2n-6	0.00	0.43	-0.01	0.00
Fatty fish intake	C18:3n-6	0.00	0.42	0.00	0.00
Fatty fish intake	C18:3n-3	0.00	0.01	0.00	0.00
Fatty fish intake	C20:1n-9	0.00	<0.001	0.00	0.00
Fatty fish intake	C20:2n-6	0.00	0.23	0.00	0.00
Fatty fish intake	C20:3n-9	0.00	0.03	0.00	0.00
Fatty fish intake	C20:3n-6	0.00	0.60	0.00	0.00
Fatty fish intake	C20:4n-6	0.00	0.62	0.00	0.00
Fatty fish intake	C20:3n-3	0.00	0.001	0.00	0.00
Fatty fish intake	C22:0	0.00	0.46	0.00	0.00
Fatty fish intake	C22:1t	0.00	<0.001	0.00	0.00
Fatty fish intake	C20:5n-3	0.00	<0.001	0.00	0.00
Fatty fish intake	C22:4n-6	0.00	0.07	0.00	0.00
Fatty fish intake	C22:5n-6	0.00	0.35	0.00	0.00
Fatty fish intake	C22:5n-3	0.00	<0.001	0.00	0.00
Fatty fish intake	C22:6n-3	0.00	<0.001	0.00	0.00
Fatty fish intake	SFA	-0.01	0.29	-0.02	0.00
Fatty fish intake	PUFA	0.00	0.86	-0.01	0.01

Exposure	Outcome[2]	β-Coefficient	P-value[3]	L95[4]	U95[5]
Fatty fish intake	n-6 PUFA	0.00	0.37	-0.01	0.00
Fatty fish intake	n-3 PUFA	0.00	<0.001	0.00	0.01
Fatty fish intake	MUFA	0.00	0.23	0.00	0.01
Fatty fish intake	Trans FA	0.00	0.78	0.00	0.00
Fatty fish intake	SFA/PUFA	0.00	0.36	0.00	0.00
Fatty fish intake	n-6/n-3 PUFA	-0.02	<0.001	-0.02	-0.01
Fatty fish intake	MCFA	0.00	0.66	-0.01	0.01
Fatty fish intake	LC-PUFA	0.00	<0.001	0.00	0.00
Mode of delivery[13]	C8:0	0.02	0.01	0.00	0.04
Mode of delivery	C10:0	0.04	0.32	-0.04	0.11
Mode of delivery	C12:0	0.25	0.15	-0.09	0.58
Mode of delivery	C13:0	0.00	0.76	-0.01	0.01
Mode of delivery	C14:0	0.31	0.04	0.02	0.59
Mode of delivery	C14:1	0.00	0.97	-0.01	0.01
Mode of delivery	C15:0	0.00	0.69	-0.02	0.02
Mode of delivery	C15:1	0.00	0.53	-0.01	0.00
Mode of delivery	C16:0	0.20	0.25	-0.14	0.55
Mode of delivery	C16:1t	0.00	0.37	0.00	0.01
Mode of delivery	C16:1n-7	0.04	0.50	-0.08	0.16
Mode of delivery	C17:0	0.00	0.82	-0.01	0.01
Mode of delivery	C18:0	-0.07	0.60	-0.33	0.19
Mode of delivery	C18:1t	-0.02	0.44	-0.05	0.02
Mode of delivery	C18:1n-9	-0.16	0.54	-0.68	0.36
Mode of delivery	C18:1n-7	0.03	0.22	-0.02	0.07
Mode of delivery	C18:2n-6	-0.59	0.02	-1.11	-0.08
Mode of delivery	C18:3n-6	0.00	0.85	-0.01	0.01
Mode of delivery	C18:3n-3	-0.03	0.34	-0.09	0.03
Mode of delivery	C20:1n-9	-0.01	0.44	-0.03	0.01
Mode of delivery	C20:2n-6	0.01	0.38	-0.02	0.05
Mode of delivery	C20:3n-9	0.00	0.22	0.00	0.00
Mode of delivery	C20:3n-6	0.01	0.15	0.00	0.03
Mode of delivery	C20:4n-6	0.01	0.16	0.00	0.03
Mode of delivery	C20:3n-3	0.00	0.32	0.00	0.00
Mode of delivery	C22:0	0.00	0.61	-0.01	0.01
Mode of delivery	C22:1t	0.00	0.87	-0.01	0.01
Mode of delivery	C20:5n-3	-0.01	0.05	-0.03	0.00

Exposure	Outcome[2]	β-Coefficient	P-value[3]	L95[4]	U95[5]
Mode of delivery	C22:4n-6	0.01	0.04	0.00	0.01
Mode of delivery	C22:5n-6	0.00	0.71	0.00	0.00
Mode of delivery	C22:5n-3	0.00	0.43	-0.02	0.01
Mode of delivery	C22:6n-3	-0.04	0.11	-0.08	0.01
Mode of delivery	SFA	0.75	0.05	0.01	1.48
Mode of delivery	PUFA	-0.63	0.03	-1.22	-0.05
Mode of delivery	n-6 PUFA	-0.55	0.04	-1.09	-0.02
Mode of delivery	n-3 PUFA	-0.08	0.10	-0.18	0.02
Mode of delivery	MUFA	-0.10	0.73	-0.68	0.48
Mode of delivery	Trans FA	-0.01	0.55	-0.05	0.03
Mode of delivery	SFA/PUFA	0.21	0.01	0.05	0.38
Mode of delivery	n-6/n-3 PUFA	-0.12	0.54	-0.50	0.26
Mode of delivery	MCFA	0.61	0.07	-0.05	1.28
Mode of delivery	LC-PUFA	-0.03	0.52	-0.10	0.05
Maternal age[14]	C8:0	0.00	0.67	0.00	0.00
Maternal age	C10:0	0.00	0.59	0.00	0.01
Maternal age	C12:0	0.00	0.78	-0.03	0.02
Maternal age	C13:0	0.00	0.21	0.00	0.00
Maternal age	C14:0	0.00	0.78	-0.02	0.03
Maternal age	C14:1	0.00	0.82	0.00	0.00
Maternal age	C15:0	0.00	0.01	0.00	0.00
Maternal age	C15:1	0.00	0.31	0.00	0.00
Maternal age	C16:0	-0.02	0.15	-0.05	0.01
Maternal age	C16:1t	0.00	0.003	0.00	0.00
Maternal age	C16:1n-7	-0.02	0.001	-0.03	-0.01
Maternal age	C17:0	0.00	0.21	0.00	0.00
Maternal age	C18:0	-0.02	0.06	-0.04	0.00
Maternal age	C18:1t	-0.01	0.001	-0.01	0.00
Maternal age	C18:1n-9	0.00	0.93	-0.04	0.04
Maternal age	C18:1n-7	-0.01	0.001	-0.01	0.00
Maternal age	C18:2n-6	0.04	0.06	0.00	0.08
Maternal age	C18:3n-6	0.00	0.43	0.00	0.00
Maternal age	C18:3n-3	0.01	0.03	0.00	0.01
Maternal age	C20:1n-9	0.00	<0.001	0.00	0.01
Maternal age	C20:2n-6	0.00	0.74	0.00	0.00
Maternal age	C20:3n-9	0.00	0.04	0.00	0.00

Exposure	Outcome[2]	β-Coefficient	P-value[3]	L95[4]	U95[5]
Maternal age	C20:3n-6	0.00	0.36	0.00	0.00
Maternal age	C20:4n-6	0.00	0.01	0.00	0.00
Maternal age	C20:3n-3	0.00	0.03	0.00	0.00
Maternal age	C22:0	0.00	0.07	0.00	0.00
Maternal age	C22:1t	0.00	<0.001	0.00	0.00
Maternal age	C20:5n-3	0.00	<0.001	0.00	0.01
Maternal age	C22:4n-6	0.00	0.02	0.00	0.00
Maternal age	C22:5n-6	0.00	0.12	0.00	0.00
Maternal age	C22:5n-3	0.00	<0.001	0.00	0.00
Maternal age	C22:6n-3	0.01	<0.001	0.01	0.01
Maternal age	SFA	-0.03	0.25	-0.09	0.02
Maternal age	PUFA	0.06	0.01	0.01	0.11
Maternal age	n-6 PUFA	0.04	0.09	-0.01	0.08
Maternal age	n-3 PUFA	0.02	<0.001	0.01	0.03
Maternal age	MUFA	-0.02	0.41	-0.07	0.03
Maternal age	Trans FA	0.00	0.005	-0.01	0.00
Maternal age	SFA/PUFA	-0.02	0.02	-0.03	0.00
Maternal age	n-6/n-3 PUFA	-0.07	<0.001	-0.10	-0.04
Maternal age	MCFA	0.00	0.96	-0.05	0.06
Maternal age	LC-PUFA	0.01	<0.001	0.01	0.02
Cod liver oil intake[15]	C8:0	0.00	0.69	0.00	0.00
Cod liver oil intake	C10:0	0.00	0.45	0.00	0.00
Cod liver oil intake	C12:0	0.00	0.97	0.00	0.00
Cod liver oil intake	C13:0	0.00	0.07	0.00	0.00
Cod liver oil intake	C14:0	0.00	0.11	0.00	0.00
Cod liver oil intake	C14:1	0.00	0.30	0.00	0.00
Cod liver oil intake	C15:0	0.00	<0.001	0.00	0.00
Cod liver oil intake	C15:1	0.00	0.002	0.00	0.00
Cod liver oil intake	C16:0	0.00	0.29	0.00	0.00
Cod liver oil intake	C16:1t	0.00	0.80	0.00	0.00
Cod liver oil intake	C16:1n-7	0.00	0.60	0.00	0.00
Cod liver oil intake	C17:0	0.00	0.02	0.00	0.00
Cod liver oil intake	C18:0	0.00	0.77	0.00	0.00
Cod liver oil intake	C18:1t	0.00	0.18	0.00	0.00
Cod liver oil intake	C18:1n-9	0.00	0.01	0.00	0.00
Cod liver oil intake	C18:1n-7	0.00	0.77	0.00	0.00

Exposure	Outcome[2]	β-Coefficient	P-value[3]	L95[4]	U95[5]
Cod liver oil intake	C18:2n-6	0.00	0.70	0.00	0.00
Cod liver oil intake	C18:3n-6	0.00	0.21	0.00	0.00
Cod liver oil intake	C18:3n-3	0.00	0.79	0.00	0.00
Cod liver oil intake	C20:1n-9	0.00	<0.001	0.00	0.00
Cod liver oil intake	C20:2n-6	0.00	0.25	0.00	0.00
Cod liver oil intake	C20:3n-9	0.00	<0.001	0.00	0.00
Cod liver oil intake	C20:3n-6	0.00	0.01	0.00	0.00
Cod liver oil intake	C20:4n-6	0.00	0.004	0.00	0.00
Cod liver oil intake	C20:3n-3	0.00	0.74	0.00	0.00
Cod liver oil intake	C22:0	0.00	0.37	0.00	0.00
Cod liver oil intake	C22:1t	0.00	<0.001	0.00	0.00
Cod liver oil intake	C20:5n-3	0.00	<0.001	0.00	0.00
Cod liver oil intake	C22:4n-6	0.00	<0.001	0.00	0.00
Cod liver oil intake	C22:5n-6	0.00	0.02	0.00	0.00
Cod liver oil intake	C22:5n-3	0.00	<0.001	0.00	0.00
Cod liver oil intake	C22:6n-3	0.00	<0.001	0.00	0.00
Cod liver oil intake	SFA	0.00	0.21	0.00	0.00
Cod liver oil intake	PUFA	0.00	0.54	0.00	0.00
Cod liver oil intake	n-6 PUFA	0.00	0.50	0.00	0.00
Cod liver oil intake	n-3 PUFA	0.00	<0.001	0.00	0.00
Cod liver oil intake	MUFA	0.00	0.03	0.00	0.00
Cod liver oil intake	Trans FA	0.00	0.63	0.00	0.00
Cod liver oil intake	SFA/PUFA	0.00	0.95	0.00	0.00
Cod liver oil intake	n-6/n-3 PUFA	0.00	<0.001	-0.01	0.00
Cod liver oil intake	MCFA	0.00	0.42	0.00	0.00
Cod liver oil intake	LC-PUFA	0.00	<0.001	0.00	0.00

[1] Models run in multiply imputed data set (n=789) in 10 imputed sets. Each predictor-percent composition model was adjusted for an indivudal set of confounders.
[2] Abbreviations are as follows: SFA = saturated fatty acid; MUFA = monounsaturated fatty acid; PUFA = polyunsaturated fatty acid; trans-FA = trans fatty acid; MCFA = medium chain fatty acid; and LC-PUFA = long chain polyunsaturated fatty acid. Outcome was percent composition of each triglyceride fatty acid, with the exception of the SFA/PUFA ratio and n-6/n-3 PUFA ratio.
[3] Values of 0.000 represent a p-value less than 0.001.
[4] Vales of 0.00 represent a lower 95% confidence interval (CI) value of <0.01.
[5] Values of 0.00 represent an upper 95% CI value of <0.01.
[6] Defined as BMI < 18.5 kg/m^2. Model adjusted for maternal age, education, parity, infant age at milk collection, formula introduction.
[7] Defined as BMI ≥30 kg/m^2. Model adjusted for maternal age, education, parity, infant age at milk collection, formula introduction.
[8] Defined by BMI according to guidelines set forth by Institutes of Medicine: for underweight women, >18.1kg; for normal weight women, >15.9kg; for overweight women, >11.3kg; and for obese women, >9.1kg. Model adjusted for gestational age, maternal age, education, parity, infant age at milk collection, formula introduction.

[9] Defined as primiparous or multiparous. Model adjusted for maternal age, education, infant age at milk collection, formula introduction.
[10] Defined as current smoker versus former/never smoker at the start of pregnancy. Model adjusted for maternal education, infant age at milk collection, formula introduction.
[11] Measured in days. Model adjusted for BMI, smoking, parity, infant age at milk collection, formula introduction.
[12] Defined as number of fatty fish dinners consumed in the previous year. Model adjusted for BMI, education, cod liver oil, infant age at milk collection, formula introduction.
[13] Defined as vaginal delivery versus cesarean section. Model adjusted for maternal age, BMI, birthweight, gestational age, excess weight gain in pregnancy, parity, smoking, infant age at milk collection, formula introduction.
[14] Defined as maternal age in years at the start of pregnancy. Model adjusted for parity, infant age at milk collection, formula introduction.
[15] Defined as servings of cod liver oil consumed in the previous year. Model adjusted for education, BMI, fatty fish intake, infant age at milk collection, formula introduction.

Supplemental Table S4. Associations[1] between select[2] triglyceride fatty acids and failure to thrive[3]

Fatty Acid[4]		Unadjusted model			Adjusted model[5]		
		Odds ratio	Confidence Interval	P-value	Odds ratio	Confidence Interval	P-value
C12:0	Lauric acid	0.99	0.89 – 1.09	0.78	1.00	0.87 – 1.14	0.96
C14:0	Myristic acid	1.03	0.92 – 1.15	0.59	1.04	0.89 – 1.23	0.62
Saturated fatty acids[6]		1.02	0.97 – 1.06	0.46	1.03	0.96 – 1.09	0.45
Saturated fatty acids/ Polyunsaturated fatty acids[7]		1.14	0.94 – 1.38	0.18	1.10	0.84 – 1.45	0.49
Medium chain fatty acids[8]		1.00	0.95 – 1.05	1.00	1.01	0.94 – 1.08	0.84

[1] Analysis run in the complete case set (n=789).
[2] Triglyceride fatty acids included in this analysis were ones that had notable inverse associations with rapid growth in previous analyses.
[3] Defined as change in weight-for-age *z*-score $\leq$-0.67 between 0 and 6 months. In the complete case set, 191 infants (24.2%) met this criteria.
[4] Exposure was the percent composition of the triglyceride fatty acid.
[5] Models adjusted for maternal age, smoking, education, pre-pregnancy body mass index, gestational weight gain, parity, and child sex.
[6] Includes C8:0, C10:0, C12:0, C13:0, C14:0, C15:0, C16:0, C17:0, C18:0, and C22:0.
[7] Exposure was the ratio, not percent composition. Polyunsaturated fatty acids included C18:3n-3, C20:3n-3, C20:5n-3, C22:5n-3, C22:6n-3, C18:2n-6, C18:3n-6, C20:2n-6[4], C20:3n-6, C20:4n-6, C22:4n-6, C22:5n-6, and C20:3n-9.
[8] Includes C8:0, C10:0, C12:0, and C14:0.
